# The Role of Colchicine in Plant Breeding

**DOI:** 10.3390/ijms26146743

**Published:** 2025-07-14

**Authors:** Baljinder Singh, Sunyoung Yun, Yeji Gil, Myoung-Hwan Park

**Affiliations:** 1 Convergence Research Center, Nanobiomaterials Institute, Sahmyook University, Seoul 01795, Republic of Korea; vishalmasih94@gmail.com; 2 Department of Chemistry and Life Science, Sahmyook University, Seoul 01795, Republic of Korea; iopbnm395648@gmail.com (S.Y.); dbwjdddsk@naver.com (Y.G.)

**Keywords:** colchicine, plant breeding, ethyl methanesulfonate, methyl methanesulfonate, chromosome doubling

## Abstract

Colchicine, a strong antimitotic drug produced by the crocus *Colchicum autumnale*, induces polyploidy by interfering with spindle formation during mitosis, making it a crucial tool in plant breeding. In this review, we give a comprehensive overview of the function of colchicine in plant enhancement, emphasizing its modes of action, application techniques, and effects on phytochemistry, physiology, and plant morphology. A wide variety of plant species, especially medicinal plants, have been studied in this context, utilizing in vitro, ex vitro, and in vivo methods for applying colchicine. In addition, we discuss the safety and effectiveness of colchicine in comparison to other polyploidy-inducing drugs, including oryzalin, trifluralin, and mutagens such as ethyl methanesulfonate and methyl methanesulfonate. Furthermore, the effects of colchicine on genetic stability and secondary metabolite production are discussed, with a focus on its usefulness in boosting the medicinal and economic potential of the target species. This synthesis highlights the ongoing use of colchicine in plant breeding and provides useful information and suggestions for future advancements in crop development via induced polyploidy.

## 1. Introduction

Colchicine, a naturally occurring tropolone alkaloid derived mostly from the autumn crocus (*Colchicum autumnale*), has long been an important compound in medicine and the study of plant biology [1,2]. It has long been used in the treatment of gout and familial Mediterranean fever due to its anti-inflammatory activity. More recently, the discovery of its antimitotic qualities has made it a vital component in cytogenetics and plant breeding [3,4]. Chromosome doubling results from the binding of colchicine to tubulin, a structural protein that creates microtubules and interferes with spindle fiber formation during mitosis [2,5]. This characteristic of colchicine makes it especially useful in stimulating polyploidy (numerous sets of chromosomes) and, consequently, the breeding of plant lines with advantageous features [6,7]. For decades, polyploidy has been used as a strategy to improve plants; new opportunities for agricultural development have been made possible through artificial chromosomal doubling [8,9]. Compared to their diploid counterparts, polyploid plants frequently exhibit larger and thicker leaves, higher biomass, better floral traits, larger cells, and higher tolerance to environmental stress [10,11]. Improved phytochemical profiles have also been observed in many polyploid taxa, including higher concentrations of secondary metabolites, such as flavonoids, alkaloids, terpenoids, and essential oils [12,13]. Secondary metabolites are frequently the main bioactive substances that provide therapeutic value of medicinal and aromatic plants; thus, these biochemical alterations are significant [14,15].

The practical applications of colchicine include various breeding goals in numerous cultivated plant species [16]. Colchicine has been used to induce polyploidy in vitro, ex vitro, and in vivo systems with differing degrees of success [10,17]. The effectiveness of colchicine treatment depends on several variables, including concentration, exposure duration, explant type, and intrinsic sensitivity of the plant species [18,19]. Colchicine therapies must be carefully balanced to achieve a level of activity that does not cause cytotoxicity but successfully induces chromosomal doubling; extended exposure or high concentrations frequently result in morphological defects and decreased viability of the plant [20,21]. Other antimitotic drugs, such as oryzalin, trifluralin, and amiprophos-methyl, have become viable substitutes for colchicine, as their polyploidization effectiveness is equivalent or better than that of colchicine, with comparatively lower toxicity in humans. Although the processes and results of mutagenic agents, such as ethyl methanesulfonate (EMS) and methyl methanesulfonate (MMS), are fundamentally different from those of colchicine-induced polyploidy, they have been used to produce genetic diversity through point mutations [22,23]. To select the best tactics for certain breeding objectives, a comparative analysis of these agents is necessary.

**Figure 1:**
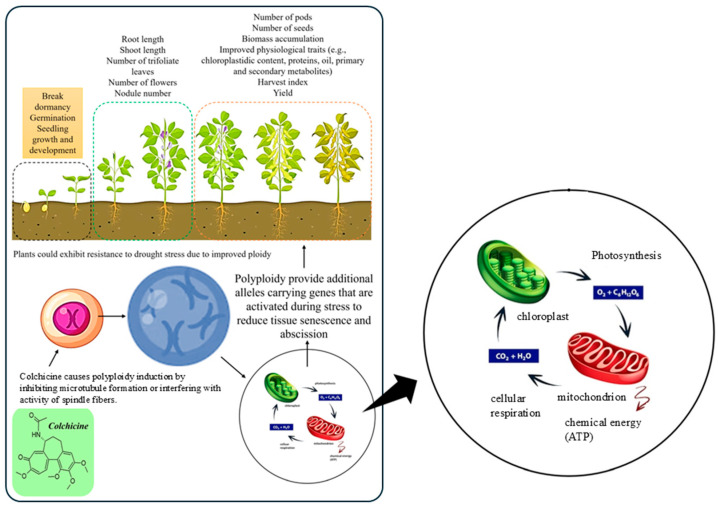
Demonstration of the potential role of colchicine in regulating the growth and development of leguminous crops. Reproduced with permission from [24], CC-BY license, Copyright © 2023, the authors. Licensee MDPI, Basel, Switzerland.

Here, we review the function of colchicine in plant breeding, focusing on how it induces polyploidy in a wide range of crop species (Figure 1) [24]. The effectiveness of numerous approaches for the application of colchicine in diverse systems has been examined. We compare colchicine with other chemical agents in terms of mechanism, safety, effectiveness, and species-specific reactions. Furthermore, a comprehensive analysis of the effects of colchicine on secondary metabolite synthesis and the genetic stability of the induced polyploids was conducted, with a focus on medicinal plants for which the yield of bioactive compounds is the primary breeding aim. In our analysis, we emphasize the varied and changing uses of colchicine in contemporary plant breeding and its potential to aid in the development of novel cultivars with increased agronomic, medicinal, and industrial value. The information presented and discussed here will be useful to researchers and breeders who wish to fully utilize colchicine in plant improvement initiatives.

This review was developed to investigate and evaluate the application of colchicine in plant breeding, emphasizing its mechanisms of action, methods of application, and relative efficacy in comparison to other agents that induce polyploidy. We also point to new developments in colchicine-mediated enhancements of the characteristics of medicinal plants. The main idea is to provide comparative and useful insights for contemporary plant breeding initiatives by combining previous research findings.

## 2. Comparison of Colchicine with Other Agents

Colchicine has historically been used to induce polyploidy in plants because of its capacity to interfere with the production of mitotic spindles. However, a number of substitute drugs, each with unique modes of action, safety profiles, and species-specific results, have surfaced as promising alternatives, including oryzalin, trifluralin, and EMS. The methods of action, effectiveness, and common applications of these drugs are contrasted with colchicine in this section. Studies that explicitly compare these substances are emphasized, offering a nuanced perspective on how scientists might select suitable mutagens according to plant species, breeding objectives, and resource availability.

Because colchicine can interfere with spindle formation during cell division, it is a key component of polyploidy in plants. Alternative treatments with different safety and efficacy levels, such as oryzalin, trifluralin, EMS, and MMS, have been developed.

### 2.1. Agents with Similar Mechanism to Colchicine

Colchicine causes polyploidy by interfering with the synthesis of microtubules, whereas dinitroaniline herbicides, such as oryzalin and trifluralin, work similarly to plant tubulins. These substitutes are useful in some plant species because they are less toxic, and lower doses are sufficiently effective.

The first application of oryzalin in *Mentha spicata* was investigated by Bharati et al., who applied a 40 µM solution (~0.01% *w*/*v*) for 48 h in order to produce synthetic polyploidization [12]. As a result, the hexaploid spearmint plants grew faster, produced bushier shoots, and had thicker and larger leaves than their diploid counterparts. With a stunning 48.9% rise from 1.74% to 2.59% (*v*/*w*), genotype P3 stood out among the other genotypes, which showed constant or slightly reduced essential oil yields. Elevated carvone and limonene levels were found in gas chromatography-mass spectrometry (GC-MS) profiles of these hexaploids, highlighting possible uses in the food, pharmaceutical, and cosmetic sectors. Additionally, genotype P6 showed significant alterations in mineral accumulation, with the calcium and magnesium contents increasing by 18.3% and 24.1%, respectively. This underscores the manner in which oryzalin-induced polyploidy can simultaneously improve nutritional value and biomass.

**Figure 2:**
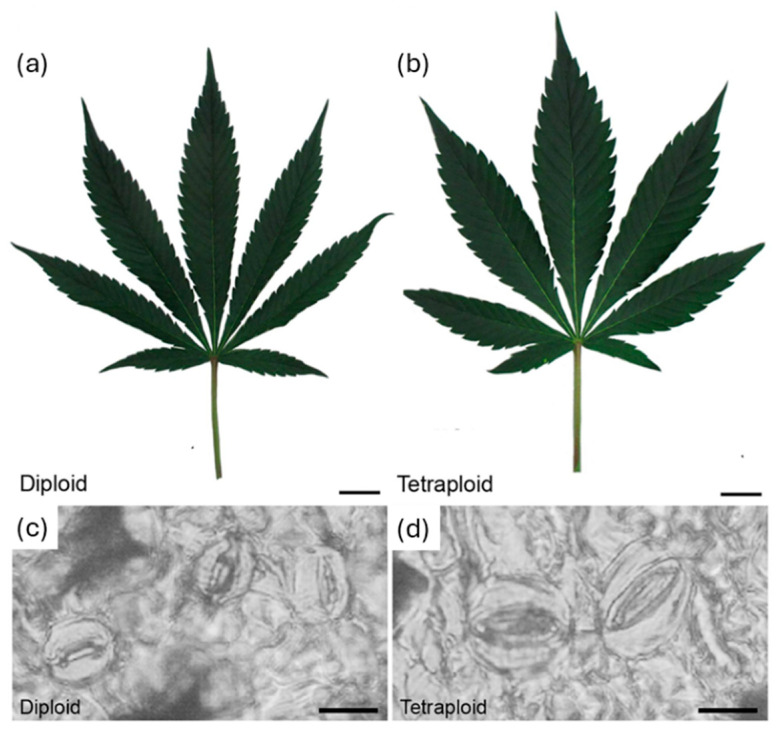
Leaf and stomata morphology. Representative images showing mature fan leaves of (**a**) diploid *Cannabis sativa* and (**b**) *C. sativa* strain 2 collected after 4 weeks of vegetative growth and 1 week under flowering lights. Scale bars, 2.5 cm. Nail polish impressions showing stomata on the abaxial surface of (**c**) diploid and (**d**) tetraploid fan leaves. Scale bars, 12 μm. Reproduced with permission from [25], CC-BY license, Copyright © 2019, the authors.

Building on this achievement, Parsons et al. created tetraploid lines with distinct morphological and biochemical characteristics by adapting the oryzalin technique to *Cannabis sativa* (Figure 2) [25]. The tetraploid cannabis had noticeably larger leaves and stomata that were around 30% larger but 46% less dense than the diploids. The terpene profiles significantly improved: floral terpenes rose by 30% and leaf terpenes by 71.5%. Sesquiterpenes with anti-inflammatory, analgesic, and antibacterial properties, like α-bisabolol and α-humulene, were especially high. While tetrahydrocannabinolic acid (THCA) stayed similar to the diploid plants, the cannabidiolic acid (CBDA) levels increased by 8.9%, while the cannabigerolic acid precursors dropped by 30%, suggesting a change in the mechanisms involved in cannabinoid production. Despite having lower initial rooting and survival rates, tetraploids grew and flowered similarly to diploids, and terpene-rich leaf extracts were as good as flower extracts, which opened up new commercial and medicinal opportunities for polyploid cannabis.

Using incredibly low doses of oryzalin (0.0000346–0.000346% *w*/*v*), Navrátilová et al. created a micropropagation strategy for the thyme cultivar “Varico” in order to provide homogenous plant material for polyploid induction [26]. In comparison to diploids, these treatments produced homogeneous tetraploids with rounder leaves, shorter internodes, and slower initial growth. The tetraploid essential oil’s greater toxicity was connected to higher amounts of trans-caryophyllene, carvacrol, thymol, and other minor components (UN3 and UN5), according to a detailed phytochemical examination. A fatal value (LD_50_) of 25.5 µg/L was demonstrated in acute toxicity tests against *Culex quinquefasciatus* larvae, demonstrating that chromosomal doubling can increase the bioactive potency of thyme.

In a greenhouse follow-up, Shmeit et al. administered 20 µM oryzalin for 24 h to 320 *T. vulgaris* explants, resulting in a 7.5% tetraploid induction rate and a survival percentage ranging from 5% to 32.5% [27]. Tetraploids that survived after a year showed a twofold increase in plant height, a 217% rise in leaf thickness, and 136–159% greater leaf dimensions. The thymol content increased from 30.31% to 48.32%, and the essential oil yield increased to 1.19% (*v*/*w*) compared to 0.81% in te diploids, with proportionate drops in precursors p-cymene and γ-terpinene. These results show that oryzalin can fine-tune both morphological and chemical traits for horticultural and industrial applications.

In vitro protocorms of the endangered orchid *Dendrobium officinale* were treated with 14.4 µM oryzalin for 24 h by Zhang et al., producing 72 stable tetraploid lines that were verified by chromosome counts and flow cytometry [28]. Despite having shorter roots and branches, these tetraploid orchids had wider labella, a feature that is highly valued in ornamental breeding. Crucially, the endopolyploidy patterns did not change the between diploid and tetraploid plants, indicating that the induced lines’ genomes were stable.

Using a trifluralin-based approach in wild blueberries (*Vaccinium duclouxii*), Lei et al. optimized a rate of 8.33% tetraploid induction with low mortality by employing 0.002% (*w*/*v*) for 48 h [29]. The tetraploid blueberries showed notable anatomical improvements, including thicker stems, larger leaves with more epidermal hairs, and increased vein thickness, upper epidermis, palisade tissue, and spongy mesophyll by 149.2%, 174.9%, and 162.4%, respectively. These results demonstrate the usefulness of trifluralin in creating strong germplasm for breeding programs.

Karami et al. used trifluralin (22.5 µM, 24 h) to create tetraploids in *Matricaria chamomilla* (German chamomile) that had expanded stomata, higher chloroplast counts, and higher levels of chlorophyll a (17.23 mg·g^−1^), chlorophyll b (4.72 mg·g^−1^), and total chlorophyll (21.95 mg·g^−1^) [19]. A chromosome number change from 2n = 18 to 2n = 36 was confirmed by cytogenetic tests, and the tetraploids’ bigger pollen grains suggested a successful reproductive transformation.

Cheng et al. achieved a 3.3% tetraploid induction rate in garlic (*Allium sativum*) by combining trifluralin (100 µM for 15 days) with plant growth regulators (6-BA and IAA). With their enlarged guard cells, reduced stomatal density, and over 95% transplant survival, these tetraploid garlic plantlets showed a viable strategy to improve this normally sterile, diploid crop [30].

**Figure 3:**
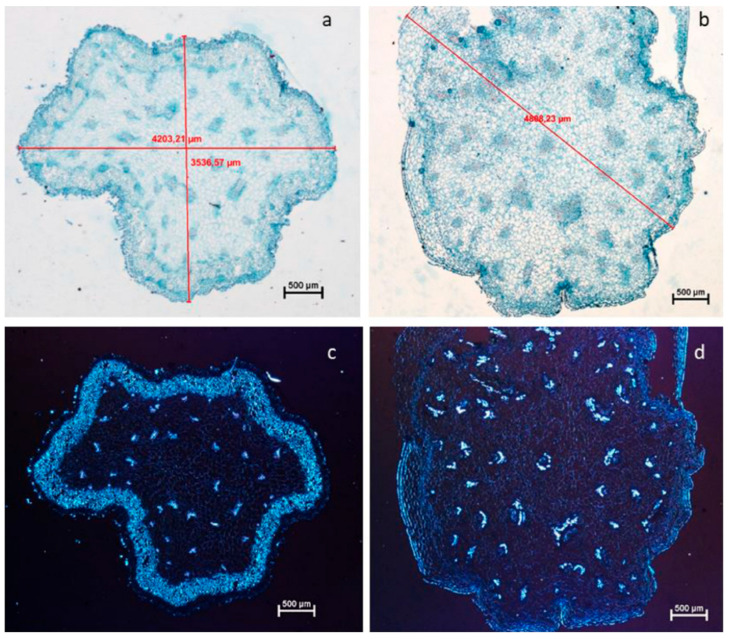
Transverse sections of flower scape: (**a**,**b**) safranine-fast green staining; (**c**,**d**) light microscope with polarization; (**a**,**c**), diploid plants with well-defined collenchyma; (**b**,**d**), tetraploids with reduced layers of collenchyma. Bars represent 500 μm. Reproduced with permission from [31], Copyright © 2018 Elsevier B.V.

In their comparison of therapies for tulip Tulipa cultivars, Podwyszyńska et al. found that trifluralin, amiprophos-methyl, oryzalin, and colchicine were the most toxic but also the most effective [31]. Stable tetraploids developed in resistant cultivars like “Fringed Black,” but they had characteristics like delayed flowering, smaller floral organs, thicker epidermal layers, and changed stem cell shape. These characteristics are difficult to overcome, but they provide new ornamental varieties (Figure 3).

Ebrahimzadeh et al. used colchicine, oryzalin, and trifluralin to assess doubled-haploid induction in *Cucumis sativus* through parthenogenesis [32]. Colchicine and trifluralin needed more careful balance to minimize toxicity and maintain viability, while oryzalin (50–75 mg/L for 18 h) proved to be the safest and most effective drug, reaching a 95% acclimation rate and robust DH production.

Talebi et al. examined colchicine, oryzalin, and trifluralin in *Agastache foeniculum* (anise hyssop) and discovered that colchicine (17,500 µM) produced 16% tetraploids (54% survival), oryzalin (100 µM) 20% (56% survival), and trifluralin (50 µM) 16% induction [3]. The resultant tetraploids had higher net photosynthesis rates (9.38 µmol CO_2_ m^−2^·s^−1^), larger leaves and flowers, more chloroplasts, improved chlorophyll indices, and increased enzyme activity (CAT, POD).

By administering 0.002% oryzalin to explants for six days, Zhang et al. were able to maximize caladium polyploid induction, attaining an induction rate of 46.67% as opposed to 15% with colchicine [33]. Larger, thicker leaves with reduced stomatal density and photosynthetic rates of 8.73 µmol CO_2_ m^−2^·s^−1^ were created by tetraploid caladiums. Along with having stronger antioxidant enzyme activity (SOD, POD, and Pro), lower relative electrical conductivity and malondialdehyde levels, and frequent chromosomal variants that are helpful for genetic study and cultivar development, these plants also demonstrated superior freezing tolerance.

### 2.2. Agents with Different Mechanisms

Chemical mutagens, such as EMS and MMS, cause point mutations by alkylating DNA bases, in contrast to colchicine, which causes polyploidy by altering chromosome segregation. These substances serve distinct functions in plant breeding programs by fostering genetic variety but do not directly cause polyploidy.

In order to isolate the RF1 mutant, Wang et al. [34] administered EMS to the local banana cultivar FJ for four hours at an LD_50_ dose of 0.8%. RF1 produced seedless fruits with creamy pulp and showed a 25.2% increase in girth and a 32% decrease in pseudostem height. Field tests conducted between 2014 and 2017 showed that RF1 flowered 24 days earlier, achieved harvest 27 days earlier, and produced fruits weighing 18.7–21.5% more than FJ across a variety of ratoon crops. The sweetness and flavor of RF1 were enhanced by its high reducing sugar-to-acid ratio (12.96), even though its overall sugar content decreased to 3.17%. The mutant also tolerated 1 °C for three days without damage, thanks to 2–8× higher soluble sugars and starch in stems and rachises, and showed enhanced resistance to Sigatoka disease, with smaller lesion sizes and longer healthy leaf spans. These attributes position RF1 as a valuable candidate for cold-tolerant, disease-resistant banana breeding.

Three husk-tomato genotypes were treated with EMS by Islam et al. [13] in order to evaluate the morphological, biochemical, and mineral alterations. In comparison to controls, mutants including C1T6, C1T7, and C2T4 showed increased plant height, fruit size, weight, and yield. Biochemical tests showed increased amounts of chlorophyll, total phenolic content, and antioxidant activity, especially in C1T3 and C2T6 mutants. With C3T6 reaching 684.3 µg/g Na and 727.6 µg/g Fe, mineral profiling revealed notable increases in sodium, magnesium, calcium, manganese, iron, copper, and potassium. These findings highlight how well EMS works to improve breeding techniques and produce nutrient-dense tomato lines.

Gao et al. [35] examined the effects of seed and microspore treatments on EMS mutagenesis in Chinese cabbage. Through microspore mutagenesis, 1034 double-haploid (DH) plants were created from 1339 embryoids and 1268 regenerants. Changes in leaf morphology, head formation, and fertility were observed in 15 stable M_1_ mutants (1.2% mutation rate). After screening 7800 seeds using seed-based mutagenesis, 701 M_2_ mutants (18.8% mutation rate) were found. These mutants included variations in head architecture, bolting time, fertility, leaf morphology (crinkling), and leaf color (complete/partial etiolation). Microspore EMS offers specific advantages for large-scale mutant library production and functional genomics, whereas seed EMS provides broad genetic variation. Microspore EMS produces homozygous mutants quickly.

Cowpea (*Vigna unguiculata* “Asontem”) received 0.4% EMS (LD_50_) from Opoku Gyamfi et al. [36], which led to 34% germination as opposed to 63% in the wild type. In addition to new stem coloring, growth patterns, and pod curvature, the M_1_ generation showed 17.8% germination and 74.5% survival (compared to 98.4% wild type). Mutants of the M_2_ generation had novel leaf colors, forms, and seed characteristics that were not observed in controls, such as kidney-shaped seeds and pale green leaves. EMS’s potential to increase genetic variability for cowpea improvement was demonstrated by the considerable expansion of quantitative trait ranges, including pod length (8.3–20.4 cm), seeds per pod (0–19), and pods per plant (1–71 vs. 10–34 in wild type).

The effects of EMS on pepper (Capsicum) M_1_ and M_2_ generations were evaluated by Arisha et al. [22]. With just slight phenotypic alterations, 93.1% of plants matured and 50.4% of seeds germinated in M_1_. Fourteen of the five hundred M_2_ families had chlorophyll-deficient mutants (albino, yellow, and light green); some yellow-green variants matured, whereas albinos died young. Under scanning microscopy, M_2_ also showed the ability of EMS to cause heritable trait variation by exhibiting bigger leaves with more palisade and spongy layers, altered branching, and dwarfism (less than 10 cm in height).

On Chrysanthemum cv., Din et al. [37] investigated 0.50%, 0.75%, and 1.00% EMS. All the doses decreased survival; however, 1.00% EMS continued to have the best rooting and the highest week 1 survival (82.1%). At 0.50%, the shoot length and quantity decreased, but at 1.00%, they increased by 49.4% in leaf area and 71.5 cm in height. EMS produced 60% pale pink flowers with a 70% mutation frequency while delaying blossoming. This dose-dependent reaction demonstrates how EMS can be used to produce new floral color variations in chrysanthemums.

The effects of 0.10–1.00% MMS on *Nigella sativa* were investigated by Amin et al. [5]. As MMS rose, pollen fertility decreased from 89.9% to 41.5%, plant survival decreased from 90.7% to 40.7%, and seed germination decreased from 94% to 42%. Particularly during metaphase, cytological examination showed micronuclei, bridges, laggards, chromosomal stickiness, and multivalents. MMS’s adjustable ability to produce genetic variation in black cumin is demonstrated by the fact that moderate MMS (0.25–0.75%) successfully generated mutations, while 1.00% proved to be too harmful.

Maleic hydrazide (MH) and MMS were compared on *Vicia faba* by Laskar et al. [15]. At 0.04%, MMS caused less severe chromatin bridges, stickiness, and micronuclei than MH, moderately decreased seedling length and pollen fertility, and suppressed germination by 29.4%. Moderate doses (e.g., 0.03%) increased the pods and seeds per plant, while greater doses decreased yield, and morphological alterations under MMS were gentler. According to these results, MMS is a milder mutagen for faba bean enhancement.

When chili (*Capsicum annuum*) was treated with 0.75% EMS and 1.00% MMS, Hasan et al. [38] caused alterations in the stem color, leaf form, flower characteristics, and pod features. At 0.10–0.25% (e.g., 82.6 cm height at 0.25% MMS; 250.5 g yield at 0.10% MMS), quantitative features, such as plant height, branch number, pods per plant, thousand-seed weight, and yield, peaked, but at 1.00% MMS, they began to drop. At 0.25% MMS, the chlorophyll concentration was 1.12 mg/g. According to these findings, chili agronomic qualities are maximized and negative impacts are minimized at moderate EMS/MMS levels.

The effects of 0.01–0.04% MMS on two fenugreek types (Desi Methi, Metha) were investigated by Naaz et al. [39]. Increased MMS caused dose-dependent decreases in chlorophyll, pollen fertility, germination, and survival. While low MMS increased growth and seed weight, higher dosages caused meiotic abnormalities and yield losses. This emphasizes the importance of mild MMS for fenugreek yield and trait development.

Samadi et al. [40] evaluated the morphology, gene expression, and apocarotenoids in saffron (*Crocus sativus*) in comparison to 0.025% colchicine (12 h) and EMS. Colchicine doubled the expression of ALDH, BGL, and CCD2; changed the profiles of crocetin ester, picrocrocin, and safranal; shortened the blooming period; increased corm survival; and expedited sprouting. EMS reduced survival and resulted in severe floral malformations. For saffron studies, colchicine is therefore a kinder technique, while EMS’s high mutation rate jeopardizes plant survival.

**Figure 4:**
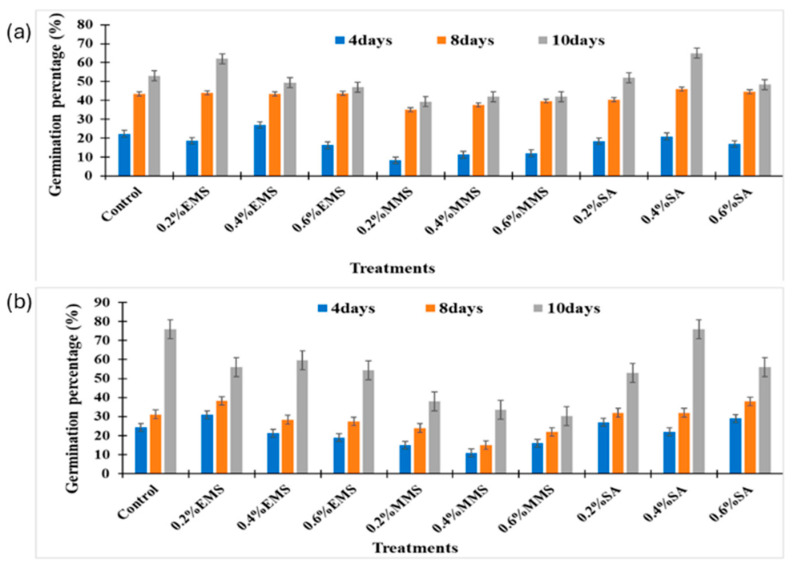
Effect of chemical mutagens on seed germination percentage (%). (**a**) Effect on SL744 soybean varieties. (**b**) Effect on SL958 soybean varieties. DAS = days after sowing. Reproduced with permission from Hamisu et al., 2024 [41], CC-BY license, Copyright © 2024, the authors. Licensee MDPI, Basel, Switzerland.

Salicylic acid (SA), EMS, and MMS were examined by Hamisu et al. [41] in relation to soybeans (SL958, SL744) (Figure 4). Chlorophyll, podding, plant height, leaf area, germination, and biochemical characteristics (protein, lipid, and fiber) were all improved by low dosages (0.2% EMS, 0.4% SA). These measures decreased with high doses (0.6% SA, 0.6% MMS). The protein peaked at 0.4% SA, while the chlorophyll peaked at 48.10 nmol/cm^2^ (0.2% EMS). Overall, the best mutagen for improving soybean quality was found to be 0.4% SA.

When Alam et al. [2] treated wheat (*Triticum aestivum*) with 0.4% EMS, MMS, SA, and colchicine, they saw dose-dependent decreases in germination (96.7→52.7%) and survival (96.7→78–81%). Stickiness, univalents, laggards, bridges, and micronuclei were among the meiotic aberrations that increased with the quantity of the mutagen; EMS created the highest aberrations and pollen sterility, whereas colchicine caused aneuploid gametes. The trade-offs between producing genetic variety and preserving reproductive viability are highlighted by these findings for each mutagen.

Cabahug et al. [42] used sodium azide, EMS, MMS, and colchicine to treat ornamental *Echeveria* (Brave, Viyant, and Snow Bunny). The two treatments that produced the most phenotypic diversity and survival were colchicine and EMS. While EMS caused more subtle alterations, colchicine significantly changed the shape, thickness, and edges of the leaves. The effects of MMS and NaN_3_ were restricted, and their survival was decreased. Overall, EMS was helpful in producing diversity with low lethality, but colchicine was most successful in producing new decorative phenotypes.

Colchicine is still the most often utilized drug for inducing polyploidy in the reviewed literature because of its efficacy and well-established methods. However, because of their greater selectivity for plant tubulins and decreased cytotoxicity, oryzalin and trifluralin have become more popular recently. Although EMS and MMS provide an alternative method through point mutation, they are more successful in producing genetic variety than in manipulating ploidy. In the end, selecting an agent requires striking a balance between the intended results, plant species sensitivity, and mutagenesis strength.

## 3. Methods of Colchicine Application

Colchicine can cause polyploidy through a variety of mechanisms, and the choice of treatment approach is crucial to its effectiveness. Ex vitro approaches are more feasible for large-scale operations, and in vitro procedures offer accuracy and regulated settings, while in vivo applications are frequently appropriate for woody and ornamental plants. Using dosage, exposure durations, and the corresponding morphological and physiological effects, this section classifies and contrasts these application techniques.

Various systems in which colchicine is used to induce polyploidy in plants are discussed in this section. Each of these techniques, which fall into three categories—in vitro, ex vitro, and in vivo—offers special benefits based on the type of plant and the intended results.

### 3.1. In Vitro System

Colchicine has been shown to cause polyploidy in Populus hopeiensis and *C. aloifolium* in sterile laboratory settings. Although high doses decreased plant survival, the treated plants displayed larger cells and changed their architecture; chromosome counts and flow cytometry were used to confirm ploidy.

Taratima et al. [21] treated protocorms in vitro under regulated environmental conditions—light/dark cycle (16/8 h), 25 ± 2 °C, and light intensity of 40 μmol∙m^−2^∙s^−1^—to examine the effect of colchicine on producing polyploidy in *C. aloifolium*. The successful induction of tetraploidy at concentrations of 0.03–0.04% colchicine over an eight-week period was verified by flow cytometry and chromosome counting. The induced tetraploid plants showed reduced overall cell density, increased stomatal and epidermal cells, and doubled DNA content. Higher doses of colchicine and longer exposure times, however, had a detrimental effect on plant development. Although colchicine successfully produced tetraploidy, careful management of treatment settings was required to avoid harmful consequences, as demonstrated by a Pearson correlation analysis, which showed that greater cell sizes were related to lower growth rates and density.

Colchicine-induced polyploidy in *Satureja khuzistanica* was studied by Shariat et al. [43], who concentrated on a variety of factors, such as plant survival, physiological and anatomical characteristics, and phytochemical production. The seedlings with developing leaflets were treated with colchicine after the seeds were germinated on ½ MS media in vitro. Tetraploid induction was the strongest at a dose of 0.05% colchicine (28.3%), and ploidy was verified by chromosomal counts and flow cytometry (2n = 4x = 60). The soluble sugars increased by 115%, total chlorophyll by 30%, phenols by 150%, flavonoids by 129%, and carotenoids by 4%, indicating notable physiological benefits for tetraploids. There was a 41% decrease in stomatal density and a 45% increase in leaf length and width, respectively. The tetraploid plants also yielded 35% more essential oil, predominantly carvacrol, showing improved biotic and abiotic resistance through polyploid induction.

A method for producing auto-octaploids from autotetraploid Populus hopeiensis was created by Wu et al. [44]. After being cultivated on MS media containing plant growth regulators, leaf explants were left in the dark for two to four days while exposed to 50–150 µM colchicine. The colchicine concentration and exposure duration affected both the survival and regeneration rates, which ranged from 30% to 86.67% for survival and from 26.67% to 76.67% for shoot regeneration. A 9-day pre-culture time and a 4-day treatment with 100 µM colchicine produced the best octaploid induction (8.61%). In accordance with gene dosage effects on cell size and division, octoploids were confirmed by flow cytometry and chromosome counting (2n = 8x = 152) and showed noticeably larger stomata, lower stomatal density, thicker leaves, shorter internodes, and slower growth rates than the diploids or tetraploids.

**Figure 5:**
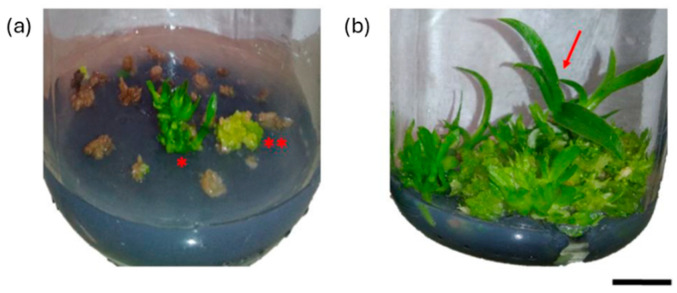
Effects of colchicine on in vitro protocorm survival of Blc. Haw Yuan Beauty × Blc. Goldenzelle “LC”: (**a**) protocorms cultured in MS medium for 120 days after treatment with colchicine at a concentration of 1000 µM and exposure time of 24 h, showing dead tissues, regeneration of PLBs **, and plantlets *; (**b**) control treatment with protocorms in plant regeneration (red arrow). Scale bar = 1 cm. Reproduced with permission from Vilcherrez-Atoche, 2023 [17], CC-BY license, Copyright © 2023, the authors. Licensee MDPI, Basel, Switzerland.

Polyploidization in the seeds and protocorms of the hybrid orchid (Blc. Haw Yuan Beauty × Blc. Goldenzelle “LC”) was investigated in vitro by Vilcherrez-Atoche et al. (Figure 5) [17]. Treatments with 500–1000 µM colchicine for 6–24 h resulted in higher rates of polyploidy, especially in protocorms (16.43%) as opposed to seeds (3.21%). Higher concentrations or longer exposure times boosted plantlet regeneration while decreasing germination and fresh weight. Using flow cytometry and chromosome counting, polyploidization was verified (the polyploids had 140 chromosomes compared to the diploids’ 70). The leaves of the treated plants were darker and thicker. The best induction of polyploidy was obtained with 500 µM colchicine for 6 h or 1000 µM for 18 h. Interestingly, considerable spontaneous polyploidization was also observed in untreated controls (11.5%).

By treating leaf explants with colchicine in vitro, Bhusare et al. [20] developed an effective technique to cause tetraploidy in Digitalis lanata. Explants were cultivated on MS medium supplemented with 6.81 µM TDZ and treated for 4–48 h at 25 ± 2 °C with 0–1% colchicine in liquid MS containing 0.5% DMSO. Explant survival and shoot regeneration were strongly impacted by the colchicine concentration and exposure duration. After 8 h of treatment with 0.5% colchicine, the plants produced healthy, dark-green branches with a maximum morphological variation rate of 33%, which flow cytometry proved was tetraploid. In comparison to diploid controls, the tetraploids produced more digoxin and digitoxin (1.61 and 1.73 times greater, respectively) and had larger, thicker leaves. The potential of D. lanata to produce pharmaceutically significant glycosides was demonstrated by this work, which was the first to report colchicine-induced tetraploids in the organism.

### 3.2. The Ex Vitro System

Colchicine increases the development and bioactive content of crops, such as fonio grasses (*Digitaria* spp.) and *Nigella sativa*, when applied to seeds or tissues outside sterile facilities. However, excessive doses are associated with adverse effects. Large-scale applications with restricted laboratory access are well-suited for this approach.

In order to enhance growth and yield characteristics, Nura et al. [18] administered colchicine to the seeds of five fonio (*Digitaria* spp.) accessions in the field. Following a four-hour soak in colchicine (0.1–2.0 mM), the seeds were planted in plots utilizing a randomized block design. Among all the accessions, the 0.1 mM concentration produced the best agronomic results, including increased germination, plant height, leaf size, and yield metrics, including seed production and tiller number. Every accession reacted differently: “Dinat” developed longer and more frequent spikes, “Jakah” produced more leaves and seeds, and “Jiw 2” displayed longer spikes. The benefits of higher colchicine concentrations diminished, highlighting the significance of dosage optimization.

Colchicine-induced mutagenesis in *Vigna unguiculata var. sesquipedalis* was assessed by Fathurrahman et al. [45] using four different colchicine doses (0–0.09%) and different soaking times (0–24 h). The pod number, weight, seed weight, and plant height were all markedly raised by the most successful treatment, which was 0.09% colchicine for 24 h. Despite not producing polyploidy, cytological examination showed that colchicine changed the chromosome structure, notably lengthening the arm. These modifications imply that colchicine can affect phenotypic characteristics and gene expression without changing the degree of ploidy.

Colchicine’s effects on *Nigella sativa* were investigated by Gupta et al. [1] by treating seeds with different concentrations (0.00625–0.1%) and tracking their growth in the wild. Shoot and root length, leaf area, seed weight, chlorophyll and carotenoid content, flavonoid and phenolic levels, and antioxidant capacity were all markedly improved at the 0.025% dosage. Stronger anti-inflammatory qualities, such as greater inhibition of albumin denaturation and hemolysis protection, were demonstrated therapeutically by plants treated with colchicine. These improvements most likely came from the stimulation of secondary metabolism brought on by stress.

Applying 0–0.2% colchicine directly to the tips of shoots allowed Aisyah et al. [23] to study the morphological alterations caused by colchicine in four genotypes of *Portulaca grandiflora*. Higher doses decreased the general growth characteristics, such as plant length and leaf breadth; however, some genotypes showed improvements in stem diameter and branching. The color of the leaves and stems varied slightly, but the shape and color of the flowers did not change. The study’s findings of positive correlations between morphological features demonstrated colchicine’s capacity to affect several traits at once. Overall, under treatment, the orange genotype performed the best.

By applying different concentrations of colchicine (0.5–2.5%) to the shoot tips of plantlets, Azizan et al. [46] assessed the effects of colchicine on *Stevia rebaudiana*. The organ size, leaf thickness, and stomatal density all increased as a result of colchicine’s disruption of mitotic spindles. The plant height and leaf diameters peaked at 2.0% colchicine. The stomatal density was highest in the 1.5% treatment (29% greater than controls), and the general morphology indicated increased production of stevioside, a desirable commercial characteristic. These findings show that colchicine, when applied carefully ex vitro, might enhance the growth and quality characteristics of *stevia*.

### 3.3. The In-Vivo System

In studies of *Citrus* spp., as well as orchids (*Dendrobium* spp.), in vivo colchicine treatments targeted entire plants. In addition to obvious alterations in the stomata and leaves, the treated plants displayed enhanced ploidy. This approach is especially effective in woody and ornamental species.

Ren et al. established an efficient in vivo method for tetraploid induction in monoembryonic citrus plants using the colchicine treatment of decapitated epicotyls [47]. The in vivo tetraploid induction method involved growing Fallglo mandarin seedlings at 25 °C (16 h light/8 h dark), decapitating epicotyls to form calli in darkness. The epicotyls were treated with 0.025% colchicine for 2 h after 9 d of dark pre-culture, achieving a tetraploid induction rate of 20.69% with minimal mixoploid incidence (1.72%). The tetraploid plants exhibited distinct morphological changes, including shorter height, darker green and thicker leaves, lower stomatal and oil gland densities, and larger stomata and oil glands than the diploids. The protocol was successfully applied to four additional monoembryonic citrus cultivars, with tetraploid induction rates ranging from 14.28% to 17.42%. Most induced tetraploids retained their monoembryonic nature and stable ploidy after one year of greenhouse growth, whereas some reverted to diploid or mixoploid states. An octoploid was induced but showed stunted growth and died, indicating that higher ploidy levels may hinder citrus development.

Revathi et al. focused on artificially inducing polyploidy in *Dendrobium crumenatum* through an in vivo colchicine treatment to improve floral characteristics [10]. In an in vivo system, 8 to 12 cm long plantlets of *D. crumenatum* were used as explants in the colchicine treatment. The plantlets were completely immersed in colchicine solutions at concentrations of 0.05% and 0.1% for 24, 48, 72, and 96 h, with air bubbling to prevent oxygen depletion. After treatment, the plantlets were washed thoroughly with running and sterile distilled water to remove excess colchicine and then transplanted into pots containing coconut husk chips, charcoal, and brick pieces as substrates. The survival rates decreased with increasing exposure duration, with the highest tetraploid induction (50%) observed at 0.05% colchicine treatment for 96 h. Mixoploidy was more common than tetraploidy, and a stomatal analysis revealed that the tetraploids had larger stomata and lower stomatal density than the diploids. The morphological traits, namely, leaf width, number of leaves, and pseudobulb diameter, showed significant improvements in the tetraploids. Flow cytometry confirmed the ploidy levels, and the study concluded that 0.05% colchicine for 96 h was the most effective treatment for polyploidy induction in *D. crumenatum*.

Kara et al. conducted a study on in vivo polyploid induction on seedlings of two *Vitis vinifera* cultivars [48]. In this in vivo study, polyploid induction was conducted on two cultivars of *V. vinifera*, Ekşi Kara and Trakya İlkeren, at the cotyledon stage. The seeds were stratified at +4 °C for 90 days, sown in a 2:1 peat-perlite mixture, and grown under greenhouse conditions. Colchicine solutions (0.1–0.6% *w*/*v*, dissolved in 1% DMSO) were applied as one drop to the shoot tip meristem twice daily for three consecutive days during the cotyledon expansion stage, when the apical meristem was exposed to maximize the interaction with the chemical. The shoot tip viability rates decreased with increasing colchicine doses, and morphological changes, such as shorter shoot lengths and altered stomatal properties, were observed. The stomatal size increased, whereas the stomatal density decreased, which correlated with the polyploidy levels. Chloroplast counts in the stomatal cells also increased in the polyploid genotypes. Flow cytometry analysis confirmed tetraploidy in seedlings treated with higher colchicine doses (e.g., 5 g/L for Ekşi Kara and 6 g/L for Trakya İlkeren), and mixoploidy was observed at lower concentrations. These findings align with those of other studies, demonstrating the effectiveness of colchicine in inducing polyploidy, with higher doses yielding more stable tetraploid genotypes.

In a study by McLeod et al., the authors aimed to refine techniques for inducing genome duplication in “I3” hemp using in vivo and in vitro approaches [49]. The explants, vegetative cuttings of *Cannabis sativa*, were treated with varying concentrations of colchicine (0–0.2%) or oryzalin (0–0.02%) in solutions containing 1% DMSO (pH 5.7) for 3, 6, or 12 h on a rotary shaker. After rinsing, the cuttings were rooted in a soilless potting mix under humidity domes with 24 h T5 light exposure, transplanted into pots, and grown in a greenhouse for three weeks to assess the survival rate and ploidy status. Of the 270 cuttings, 85 were rooted successfully, with a 26% polyploid induction rate, including 20 cytochimeras and two tetraploids. The colchicine treatments (0.05% and 0.2% for 12 h) yielded the most polyploids, whereas the Surflan treatments, which use oryzalin as the active herbicidal ingredient, only produced mixoploids. Higher colchicine concentrations (0.2%) generally resulted in increased polyploidy but reduced the survival rates, which is consistent with previous findings. Although the Surflan treatment showed limited success, the colchicine treatment demonstrated the potential for efficient genome doubling in hemp, particularly with longer treatment durations and higher concentrations.

Despite the distinct benefits of each application technique, in vitro systems provide the most controllable and dependable polyploidy induction. For field crops and circumstances where laboratory access is restricted, ex vitro techniques are advantageous. Even though they are frequently less accurate, in vivo systems are especially helpful for breeding initiatives aimed at decorative or perennial species. With trade-offs between survival rate and induction efficiency seen across systems, the effectiveness of each strategy mostly rests on optimizing the treatment parameters such as colchicine concentration, explant type, and exposure length.

A summary table has been included to highlight the main chemical mutagens covered in this publication in order to give a clear perspective and make comparisons across different studies easier (Table 1). The target plant species, mode of action, and main physiological or genetic consequences caused by each chemical are shown in this table. A thorough grasp of the ways in which various mutagens, including colchicine, EMS, MMS, and others, contribute to plant trait modification, polyploidy induction, and genetic variability is supported by the table’s unified format, which improves clarity. Researchers can use this synthesis as a useful guide for choosing appropriate drugs for particular breeding goals.

## 4. Colchicine in Medicinal Plant Breeding

The main purpose of colchicine-induced polyploidy in medicinal plants is to improve characteristics linked to the production of secondary metabolites, stress tolerance, and morphological quality. Examples of these applications in a variety of species are shown in this section, with a focus on the genetic and biochemical alterations seen in plants treated with colchicine. Particular focus is placed on metabolite yields, such as parthenolide, thymol, and chicoric acid, among others, which directly enhance these crops’ therapeutic potential.

In medicinal plant breeding, colchicine is frequently used to induce polyploidy by interfering with mitosis and chromosome doubling. In many species, this method can improve growth, resilience to stress, and the synthesis of bioactive compounds. Its use in genetic stability, metabolite augmentation, chromosomal doubling, and dosage optimization is highlighted in this section.

### 4.1. Applications in Chromosome Doubling

Ajowan and purple coneflower (*Echinacea purpurea*) are two medicinal plants in which colchicine treatment is successful in producing tetraploidy, resulting in enhanced growth characteristics and increased quantities of substances known to have antioxidative, antiviral, antifungal, and immunostimulatory effects, such as thymol and cichoric acid (the latter showing anti-HIV-1 activity). Through colchicine treatment of these plants, the pharmaceutical quality of these compounds and total yield is enhanced.

Abdoli et al. successfully induced tetraploidy in *E. purpurea* seedlings using a 0.25% (*w*/*v*) colchicine treatment [50]. Key indicators, such as stomatal characteristics, chloroplast count in guard cells, and leaf dimensions, were identified for the early detection of tetraploids. The tetraploid plants showed 45% and 71% increases in chicoric acid and chlorogenic acid contents, respectively, compared to the corresponding yields in the diploid plants, indicating that tetraploidy can enhance the production of secondary metabolites. These results highlight the potential of tetraploid *E. purpurea* for selective breeding and enhancing secondary metabolite production.

Majdi et al. reported a successful tetraploid induction, which could pave the way for triploid development to enhance productivity [51]. The medicinal plant feverfew, *Tanacetum parthenum*, contains parthenolide, a sesquiterpene lactone present in the aerial portion of the plant, which has anti-migraine activity. Colchicine treatment altered chromosome doubling and length, adversely affecting survival rates and initially reducing growth, owing to lower cell division. The tetraploid plants exhibited expanded stomata, trichomes, leaves, flowers, and seeds, and increased cell size, consistent with other species. These tetraploid plants demonstrated decreased stomatal density and photosynthetic efficiency but increased chlorophyll content and parthenolide synthesis, notably in the flowers. Stable tetraploidy through several generations indicates the potential for boosting parthenolide output, making it a promising technique for feverfew enhancement.

Sadat-Noori et al. achieved maximum efficiency of DNA duplication by effectively establishing an in vitro method for producing tetraploidy in ajowan (*Trachyspermum ammi*) through treatment with 0.05% colchicine for 24 h [16]. The tetraploid plants displayed improved morphological features, such as increased plant height, leaf length, stem diameter, and seed length, but lower survival rates than the diploids. While stomatal density was reduced in the tetraploids, the stomatal size increased. Tetraploidy produced 2.5 times more thymol than did the diploid controls, and it also changed the secondary metabolite profile. The concentration of thymol increased and became the dominant compound in the tetraploids, although the overall percentages of most essential oil components declined. These findings suggest that tetraploidy induction may improve the medicinal and commercial value of ajowan, as well as other plants.

### 4.2. Impact on Secondary Metabolite Production

Polyploids frequently produce larger yields of secondary metabolites. Treatment with colchicine is known to boost the level of flavonoids, essential oils, and terpenoids in certain species, such as Citrus limon and nuruozoak, *Salvia leriifolia* (Lamiaceae). Although results can differ by species, these benefits are associated with changed gene expression.

Estaji et al. demonstrated that autotetraploidy induction significantly affects the quantity and composition of essential oils in the leaves of *S. leriifolia* [52]. The plant produces bioactive compounds, such as flavonoids, phenolic acids, monoterpenes, and diterpenes, which are employed in the food, pharmaceutical, and medicinal industries. In addition, it has antibacterial, anti-inflammatory, and pain-relieving properties, making it a strong candidate for clinical testing. Among the 20 essential oil constituents found in tetraploid plants, eight were unique compounds absent from the diploid plants, whereas two compounds found in the diploid plants were absent from the tetraploid plants. The generation of secondary metabolites was influenced, enzyme activity was changed, and gene dosage was enhanced by chromosome doubling. Qualitative and quantitative differences in essential oil profiles are caused by structural and functional genomic alterations in polyploids, such as gene silencing. Tetraploids were effectively produced by treating shoot apical meristems; these plants showed altered essential oil composition and enhanced biomass.

**Figure 6:**
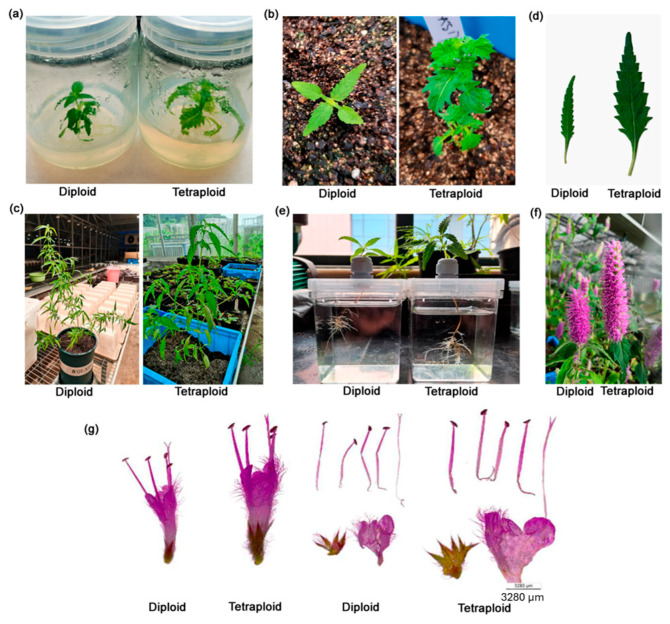
Phenotype characteristics and chromosome number of diploid and tetraploid *Elsholtzia splendens*. (**a**) Comparison of phenotype characteristics of different ploidy-level plants at tissue culture stage. (**b**) Different ploidy-level plants were transplanted into soil. (**c**) Phenotype characteristic comparison of different ploidy-level plants at adult stage. (**d**) Leaf morphology of two different ploidy-level plants at adult stage. (**e**) Tetraploid plants produced more lateral roots. (**f**) Tetraploid inflorescence displayed increased length. (**g**) Anatomical diagram comparison of one floret between diploid and tetraploid. Reproduced with permission from Liu et al., 2024 [53], CC-BY license, Copyright © 2024, the authors. Licensee MDPI, Basel, Switzerland.

Liu et al. [53] investigated how polyploidization affected secondary metabolite production in the annual herb *Elsholtzia splendens* Nakai ex F. Maek (Lamiaceae) (Figure 6). *E. splendens* is highly prized due to its remarkable ability to absorb and accumulate Cu in soils. It was first used in traditional Chinese medicine as an antiviral, antibiotic, and antispasmodic medicine, as well as a treatment for asthma. Even today, *E. splendens* is frequently used as a component of folk medicine to encourage blood flow and resolve blood stasis. Diploids and tetraploids exhibit different patterns of secondary metabolite accumulation. Compared to diploid plants, tetraploid plants produce more flavonoids and phenolic acids, including the recently discovered methoxylated flavonoids wogonin, oroxylin A, baicalin, and chrysin. Because of their selective toxicity to cancer cells, methoxyflavonoids exhibit a variety of biological actions, such as anticancer, anti-inflammatory, and neuroprotective properties. They also show promise for cancer therapy.

**Figure 7:**
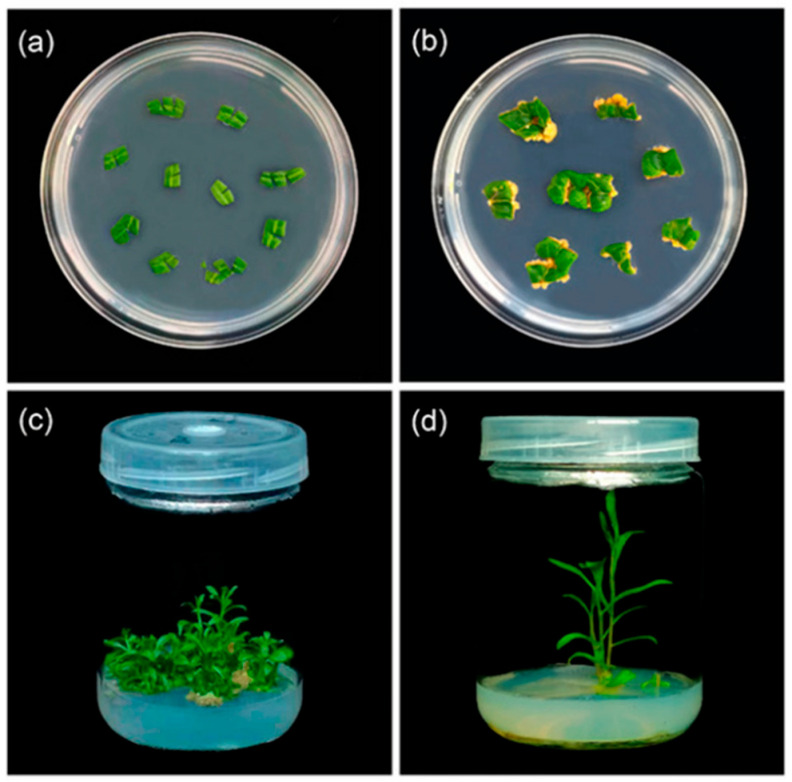
Plant regeneration from leaf explants of Lycium chinense: (**a**) leaves were transferred to MS differentiation medium with different concentrations of growth regulators; (**b**) a callus appeared at the incision; (**c**) leaves successfully regenerated adventitious shoots after 50 days; (**d**) adventitious buds rooted. Reproduced with permission from Zhang et al., 2024 [6], CC-BY license, Copyright © 2024, the authors. Licensee MDPI, Basel, Switzerland.

The first effective induction of tetraploid *Lycium chinense* plants and adventitious shoot regeneration from leaf explants using colchicine therapy was reported by Zhang et al. (Figure 7) [6]. The berries of *Lycium chinense*, known as goji berries or wolfberries, are rich in bioactive substances that prevent aging, reduce blood sugar levels, and support the liver and kidneys. After 12 days of preculture, the most effective dose of colchicine for polyploid induction was 50 mg/L for 24 h. The tetraploids outperformed the diploids in terms of biomass, water retention, leaf size, thickness, and chlorophyll content. Additionally, compared to the diploids, the tetraploids had a lower stomatal density but larger stomata, with more chloroplasts per guard cell. In terms of biochemistry, the tetraploids showed noticeably higher concentrations of crucial minerals (calcium, iron, and zinc) and bioactive substances (polysaccharides, carotenoids, phenolics, and flavonoids) than the diploids. Nevertheless, there was variation across the tetraploid strains; strain T10, for instance, had the highest polysaccharide content but the lowest amounts of carotenoids (with the exception of violaxanthin and (E/Z)-phytoene, which significantly increased). Strain T39 also showed the lowest levels of calcium and zinc but the highest iron concentration. These results underscore the fact that polyploidization does not always result in consistent increases in metabolite synthesis.

Bhuvaneswari et al. [54] developed an in vitro procedure for employing colchicine to induce tetraploidy in lemon (*Citrus limon*), a notable source of essential oils in the food, medical, and cosmetics industries, and which has demonstrated notable advancements in tetraploidy. In terms of morphology, the authors of the study reported that the tetraploids were 80% taller than the diploids, had more and longer leaves, and had a higher chloroplast density but a lower stomatal density. Although the tetraploid and diploid essential oil outputs were similar, the tetraploid oil had larger concentrations of limonene, lanceol, and β-bisabolene; in contrast, the diploid oil was found to have more (Z,E)-α-farnesene and other components not present in the tetraploids. While the MTS and STS genes remained steady, guaranteeing constant monoterpene and sesquiterpene content, gene expression studies showed that the tetraploids had significantly higher levels of LS, CHS, and PAL genes, which are associated with increased limonene production. Furthermore, because of the greater quantities of limonene and lanceol, the essential oils produced from the tetraploids showed noticeably increased antioxidant activity. These results demonstrate that polyploidization improves the morphological characteristics, biochemical makeup, and antioxidant qualities of *C. limon*.

Lin et al. used colchicine to successfully induce tetraploidy in paper mulberry, *Broussonetia papyrifera*, from the leaves, calli, and seeds [55]. The trees grow quickly and are valued for their fibrous wood and bark, which is used to make paper, as well as its potential as a high-nutrition feed for cattle. Owing to their greater tolerance and improved exposure to colchicine, the leaves exhibited the highest induction rate. In comparison to the diploids, the tetraploid plants showed shorter internodes and plant height but larger ground diameter, leaf length, width, petiole length, and stomatal size. The tetraploids were found to be anatomically more drought-resistant and photosynthetically capable because of their thicker palisades and spongy tissues, tighter leaf morphologies, and higher chlorophyll, nitrogen, and net photosynthetic rates. As in other polyploid species, tetraploid birch showed traits such as larger organs and decreased stomatal density, which enhance water retention and stress tolerance. These findings imply that tetraploid *B. papyrifera* may be useful as a drought-tolerant forage species, and further research is required to determine its nutritional value and practicality.

Kasmiyati et al. demonstrated that artificial polyploidization in the medicinal plant *Artemisa cinerea* (Compositae) enhanced the flavonoid content (quercetin and kaempferol) while impacting growth and the chlorophyll levels [56]. *A. cina* produces artemisinin, a key compound effective against drug-resistant malaria and other diseases, such as leishmaniasis, hepatitis B, and certain cancers. The authors of the study report that polyploid genotype J had the greatest dry weight of roots and shoots, whereas genotype M had the shortest plant height and slowest growth. Because of structural alterations in chloroplasts, polyploid plants possess less chlorophyll than diploids. Compared with the diploids, the polyploid plants (J and M) showed noticeably higher concentrations of flavonoids (kaempferol and quercetin). KJT (diploid) had the lowest levels of both flavonoids, whereas the M genotype had the highest quercetin content (52.92 μg/mL). In line with findings from other medicinal plants, polyploidization increased the flavonoid concentrations, most likely as a result of altered gene expression and increased allelic diversity.

### 4.3. Genetic Stability in Polyploid Plants

Colchicine-generated polyploid plants, such as Stevia rebaudiana and Mentha × villosa, have exhibited consistent characteristics throughout generations. Improved stress tolerance, metabolite levels, and morphology all indicate the potential of colchicine development in the development of stable plant lines with useful characteristics.

Moetamedipoor et al. successfully induced a hexaploid mojito mint (*M.* × *villosa*) with a duplicated chromosome number (2n = 6x = 72), resulting in significant morphological and biochemical improvements compared to its triploid counterpart [11]. Increasing the colchicine concentration and treatment duration reduced survival rates, with the highest chromosome doubling efficiency (100%) occurring at 0.05% colchicine for 9 h. The hexaploid plants exhibited thicker, darker green leaves, fewer stomata, and a 64% increase in the essential oil yield from 0.87% to 1.43%. Key phytochemicals, such as menthofuran, 1,8-cineole (eucalyptol), and pulegone, known for their respiratory health benefits, were substantially enhanced, with menthofuran (28.51%), 1,8-cineole (28.45%), and pulegone (20.14%) comprising 77.1% of the total oil composition. New compounds, like α-pinene and limonene, were also detected. These findings underscore the potential of polyploidy as an effective breeding strategy for improving essential oil production and secondary metabolite profiles and highlight hexaploid mojito mint as a promising candidate for commercial cultivation and medicinal applications.

Hamarashid et al. revealed that colchicine-induced *Stachys byzantina* polyploidy significantly enhanced the morphological, physiological, and phytochemical traits [14]. *S. byzantina* (lamb’s ear) is a medicinal plant known for its essential oils, rich in monoterpenes (linalool, α-cadinol, cubenol, and menthone), which exhibit potent antiviral, antifungal, antibacterial, and antioxidant properties. Optimal treatment (0.2% colchicine for 12 h) achieved an induction efficiency of 18%, balancing high polyploidy rates with acceptable survival. The tetraploid plants exhibited thicker, darker green leaves, wider and longer leaf dimensions, shorter stems due to reduced internode length, and larger but less dense stomata (mean stomatal frequency: 22/µm^2^ in tetraploids vs. 17/µm^2^ in diploids). Chlorophyll *a* and *b* and the total chlorophyll content significantly increased, although the carotenoid levels remained unchanged. A phytochemical analysis demonstrated notable increases in secondary metabolites, including a 30% rise in the linalool content (28.2 mg/100 g in tetraploids vs. 21.6 mg/100 g in diploids), along with higher concentrations of α-cadinol, cubenol, α-terpineol, and menthone. These enhancements were attributed to gene expression and epigenetic changes induced by polyploidization, highlighting their potential to improve morphological traits and boost phytochemical production in medicinal plants.

Talei et al. investigated colchicine-induced polyploidy in *Stevia rebaudiana* Bertoni and identified the optimal conditions for chromosome doubling [7]. *S. rebaudiana*, a medicinal plant of the Asteraceae family, is chiefly valued as a natural non-calorie sweetener. Its leaves contain potent sweet-tasting glycosides (particularly stevioside and rebaudioside-A) that are 300–400 times sweeter than sugar. The highest rate of polyploidy induction and survival rate were observed with 0.2% colchicine for 24 h; higher concentrations or longer exposure times reduced survival. The tetraploid plants exhibited significantly larger stomata than did the diploids; the larger stomata in the tetraploids suggested greater photosynthetic and transpiration capacities. The tetraploid plants exhibited higher levels of rebaudioside A, a key compound synthesized and stored in *S. rebaudiana* leaves. This increase was correlated with improved leaf morphology and enhanced stomatal characteristics. The study highlights that colchicine-induced polyploidy not only modifies plant growth and cytological traits but also boosts the production of economically valuable compounds, such as rebaudioside-A.

Zhou et al. developed a method to induce polyploidy in Fengtou ginger using colchicine [8]. Fengtou ginger, prized for its crispy, fiber-free texture, has a low overall crop yield (30,000 kg/ha), driving the need for higher-yielding commercial ginger varieties to meet growing global demand. The authors of the study found that the highest tetraploid induction rate occurred with 150 mg/L colchicine in liquid medium for 7 days. The tetraploid plants exhibited wider, thicker, and darker green leaves than did the diploids, along with larger stomata and guard cells but had lower stomatal density. In addition, the leaf area index of the tetraploids was significantly reduced, which contributed to improved dry matter quality and yield. The tetraploid plants grew more slowly during early subcultures but eventually showed no significant growth differences from the diploids after two or three subcultures. The tetraploid plants exhibited significant increases in soluble proteins, soluble sugars, and proline, which were positively correlated with stress resistance. The carotenoid content increased significantly, likely due to its sensitivity to stress. Malondialdehyde, an indicator of oxidative stress, also increased, suggesting some degree of cellular damage. The tetraploids show potential for improved stress adaptation and increased secondary metabolite content, such as carotenoids and soluble proteins, which enhance plant resilience and productivity.

### 4.4. Optimization of Dosage and Treatment Methods

The timing and dosage of colchicine treatment are crucial for successful polyploidy: overdosing can kill the plants; on the other hand, inadequate exposure can lower the efficiency of the treatment.

Yousef et al. focused on inducing polyploidy in garlic (*Allium sativum* L.) to enhance its productivity and biochemical properties [4]. The high value of garlic in nutrition and medicine is due to its unique sulfur compounds (e.g., allicin, alliin, and diallyl disulfide) and essential nutrients, such as selenium. Garlic is well known for its antimicrobial, antitumor, and immunostimulant properties. In a study by Yousef et al., tetraploid plants exhibited larger stomata; wider, thicker, and darker green leaves; and improved morphological traits, such as plant height, fresh weight, and bulb size, compared to diploids. Biochemically, the total oil content in the tetraploids was double that of the diploids (0.1% vs. 0.05%). The tetraploids had higher levels of sulfur-containing compounds in their oil, including diallyl disulfide, allyl methyl trisulfide, and diallyl trisulfide, which were 15–25% higher than those in the diploids. The tetraploids also showed higher chlorophyll, nitrogen, phosphorus, and potassium contents. Enhanced levels of secondary metabolites and higher chlorophyll, nitrogen, phosphorus, and potassium levels further supported the improved growth and metabolic activity. These findings highlight that polyploidy is a promising tool for breeding high-value garlic varieties with superior yield and medicinal potential, particularly in the pharmaceutical industry.

Sanaei-Hoveida et al. demonstrated significant improvements in the essential oil yield and composition in tetraploid cumin plants compared to those in diploids [9]. Cumin (*Cuminum cyminum* L.), an annual plant in the Apiaceae family, is renowned for its medicinal and culinary uses. Its seeds are rich in essential oils and bioactive compounds, including cuminaldehyde, cymene, and β-pinene, which give cumin its distinctive aroma, as well as its pharmacological properties, such as anticancer, antioxidant, stimulant, and carminative effects. Tetraploid induction using 0.05% colchicine was the most effective, with genotypes KBA4 and SKD6 producing the most polyploid plants. The tetraploids exhibited enhanced morphological traits, such as larger stems, roots, and seeds, leading to a higher biomass and harvest index. The essential oil yield in the tetraploids increased by 30–100% across genotypes, with KBA4 achieving the highest yield (3.5%) and YAR1 the lowest (1.95%). The tetraploids showed lower monoterpene and higher monoterpenoid percentages than the diploids, with variations in sesquiterpenes depending on the genotype. Cuminaldehyde, the key component of cumin essential oil, was significantly increased in the tetraploids, with genotype SIV8 showing the greatest increase. These findings highlight that polyploidy is a valuable technique for enhancing cumin productivity, essential oil content, and secondary metabolite composition, particularly for commercial and medicinal applications.

Among the species under investigation, such as *Mentha spicata*, *Stevia rebaudiana*, *Echinacea purpurea*, and *Nigella sativa*, treatment with colchicine consistently produced more secondary metabolites and improved morphological characteristics. The degree of these enhancements, however, differed according to species, colchicine dosage, and administration techniques. Some species, such as *Portulaca grandiflora*, exhibited genotype-specific responses, whereas *Stevia* and *Ajowan* showed notable increases in leaf size and phytochemical content. These comparative results highlight the necessity of customized methods based on the plant type and breeding goal.

Colchicine has proven to be very helpful in enhancing the pharmacological and agronomic properties of medicinal plants. After receiving therapy, many species show notable increases in biomass and the synthesis of bioactive compounds, while others show decreases in fertility or survival. Colchicine’s continuous usage in medicinal plant breeding efforts is supported by the steady improvement of particular metabolites, as well as predicted morphological changes. To balance plant health and yield, species-specific optimization is still essential.

A summary of key studies on colchicine-induced polyploidy in medicinal and crop plants is presented in Table 2 to allow for easy comparison of the concentrations, application methods, and observed phenotypic effects.

## 5. Conclusions

Colchicine has long been a fundamental tool in plant breeding because of its strong propensity to generate polyploidy, which frequently results in notable improvements in the morphology, vigor, and secondary metabolite content of plants. Despite its long history of usage, colchicine has recently attracted attention as a versatile and affordable agent, particularly for the development of therapeutic plants, thanks to developments in plant biotechnology and molecular breeding.

Colchicine-induced polyploidy regularly improves favorable features across a variety of species, including *Mentha spicata*, *Stevia rebaudiana*, *Nigella sativa*, *Echinacea purpurea*, and others, according to this review, which summarizes results from a wide range of investigations. Increased biomass, thicker leaves, larger stomata, and higher concentrations of bioactive substances, including thymol, carvone, and chicoric acid, are among the improvements. However, a number of variables, including plant genotype, explant type, concentration, exposure time, and application mode (in vitro, ex vitro, or in vivo), have a significant impact on the results of colchicine treatment. To assist with and direct the use of colchicine in various breeding scenarios, our comparative study and tabular synthesis highlight both the species-specific differences in efficacy and the common responses.

Despite its efficacy, colchicine has drawbacks, including toxicity, response unpredictability, and decreased fertility in certain polyploid plants. In order to increase survival rates and improve result predictability, it is imperative that its protocols be improved. New technologies, including real-time ploidy detection methods, genomic and metabolomic profiling, and flow cytometry, present intriguing chances to maximize colchicine use and gain a deeper comprehension of the physiological and molecular alterations brought on by polyploidization.

In the future, more accurate and effective breeding programs may result from combining colchicine-induced mutagenesis with contemporary breeding methods, like CRISPR/Cas9, transcriptome-assisted selection, and high-throughput screening. Additionally, low-toxicity analogs or delivery methods (such as nano-formulations) could be developed to lessen side effects without sacrificing effectiveness. Additionally, converting experimental success into commercial viability will require standardizing treatment parameters across species and developing cultivar-specific methods.

In summary, colchicine is still a useful and effective technique in plant breeding, especially when it comes to improving the qualities of medicinal plants. Improved, stable, and high-performing plant types could be produced with careful use and integration using contemporary molecular techniques.

## Figures and Tables

**Table 1:** Overview of colchicine and chemical mutagen alternatives used in plant improvement.

No.	Agent Type	Example(s)	Mode of Action	Key Application Outcomes	References
1	Antimitotic	Colchicine	Disrupts microtubule formation (polyploidy)	Induces polyploidy; alters morphology and secondary metabolite production	[3,20,21,43,44]
2	Dinitroanilines	Oryzalin, Trifluralin	Binds plant tubulin (polyploidy)	Safer polyploidy induction in *Mentha*, *Cannabis*, *Thymus*, *Blueberry*	[12,25,26,27,28,29,31,32,33]
3	Alkylating Agent	EMS (ethyl methanesulfonate)	Alkylates guanine (point mutations)	Trait improvement in *Banana*, *Tomato*, *Cowpea*, *Pepper*	[13,22,34,35,36,37]
4	Alkylating Agent	MMS (methyl methanesulfonate)	Induces chromosomal aberrations	Morphological and physiological variation in *Nigella*, *Chili*, *Fenugreek*	[5,15,38,39]
5	Sulfonic Acid Deriv.	SA (sodium azide)	Produces point mutations via base alteration	Enhances protein, lipid, and fiber traits in soybean	[41]

**Table 2:** Summary of colchicine-induced polyploidy across various plant species.

No.	Plant Species	Colchicine Conc. and Time	Application Method	Observed Effects	References
1	*Nigella sativa*	0.025%, 8 h	Seed soaking	↑ shoot/root length, flavonoids, antioxidants, chlorophyll, phenolics	[1]
2	*Cuminum cyminum* (cumin)	0.05%	In vitro or seed-based	↑ essential oil yield (30–100%), ↑ cuminaldehyde, stem/root/seed size	[9]
3	*Mentha spicata*	40 µM, 48 h	In vitro (oryzalin)	Tetraploids showed increased leaf area, bushiness, and carvone/limonene content	[12]
4	*Stachys byzantina*	0.2%, 12 h	In vitro	↑ linalool, chlorophyll, leaf size, ↓ stomatal density; 18% polyploidy rate	[14]
5	*Portulaca grandiflora*	0.0–0.2% (drops), 3 days shading	Shoot tip (ex vitro)	↑ stem diameter, branch number, leaf morphology; genotype-specific variation	[23]
6	*Satureja khuzistanica*	0.05%, 4 days	In vitro leaf explants	↑ chlorophyll, sugar, phenols; ↓ stomatal density; tetraploid confirmed by flow cytometry	[43]
7	*Stevia rebaudiana*	1–2.5%, 48 h	Shoot tip (ex vitro)	Larger leaves, altered margins, ↑ stevioside, ↑ stomatal size/density	[46]
8	*Citrus* spp.	0.025%, 2 h	Decapitated epicotyls	↑ leaf thickness, ↓ oil gland/stomatal density, 20% tetraploid induction	[47]
9	*Vitis vinifera*	1–6 g/L, 3 days	Cotyledon shoot tip	↑ stomatal size, ↓ density; tetraploids confirmed; viability dose-dependent	[48]
10	*Echinacea purpurea*	0.25%	Seedlings	↑ chicoric/chlorogenic acid content, larger leaves, tetraploidy achieved	[50]

In the table, the arrows (↑ and ↓) are used as shorthand to indicate increases or decreases in observed biological traits after colchicine or antimitotic treatment.

## Data Availability

Not applicable.
